# AdipoGauge software for analysis of biological microscopic images

**DOI:** 10.1080/21623945.2020.1787583

**Published:** 2020-07-11

**Authors:** Mohammad Yosofvand, Sanka Liyanage, Nishan S. Kalupahana, Shane Scoggin, Naima Moustaid-Moussa, Hanna Moussa

**Affiliations:** a Department of Mechanical Engineering, Texas Tech University, Lubbock, TX, USA; b Department of Physiology, University of Peradeniya, Peradeniya, Sri Lanka; c Department of Nutritional Sciences, Texas Tech University, Lubbock, TX, USA; d Obesity Research Institute, Texas Tech University, Lubbock, TX, USA

**Keywords:** Adipose Tissue, obesity, histology, image Processing, microscopic image analysis

## Abstract

Obesity is a complex disease of global epidemic proportions. Adipose tissue expansion and chronic low-grade inflammation, locally and systemically, are hallmark features of obesity. Obesity is associated with several other chronic diseases, which are also characterized by inflammation. Determination of adipocyte size and macrophage content in adipose tissue is a critical step in assessing changes in this tissue with obesity. Here, we introduce a complete standalone software package, **AdipoGauge**, to analyse microscopic images derived from haematoxylin and eosin (H&E)-stained and immunofluorescently stained histology sections of adipose tissue. The software package is a user-friendly application that does not require a vast knowledge of computer science or costly commercial tools. AdipoGauge includes analysing tools that are capable of cell counting and colour separation. Furthermore, it can quantify the cell data in images both with and without clear boundaries around the cells. It can also remove objects from the image that are not intended for analysis, such as blood vessels or partial cells at edges of slide sections. The simple and state-of-the-art graphical user interface requires minimal time and learning.

## 1. Introduction

^1^ Obesity is a complex disease that has reached epidemic proportions in the U.S and worldwide [1,2] and is characterized by expansion of white adipose tissue (WAT). This chronic, low-grade inflammatory disease involves infiltration of macrophages into the WAT, which leads to increased production and secretion of pro-inflammatory adipocytokines from adipose tissue and reduced anti-inflammatory adipokines [3,4]. These cytokines affect metabolism of other tissues and contribute to systemic inflammation and insulin resistance [5]. Enlarged adipocytes (hypertrophy), increased adipocyte number (hyperplasia) and increased macrophage content in adipose tissue are important characteristics in adipose tissue expansion. All these features are typically examined in order to estimate changes that occur in adipose tissue with obesity, whether dietary or genetic, and to assess the effects of various dietary or pharmacological anti-obesity and anti-inflammatory interventions [6,7]. Thus far, most researchers have relied on manual measurements (counting cells by hand), or publicly available software, such as NIH ImageJ, for analyses of histology sections of adipose tissue [8]. While these tools have been very useful, both approaches are tedious and time consuming, and justify the need for an automated and rapid means to measure various cellular features in adipose tissue histological sections.

The first attempts to determine adipose cell size started in the late 1960 s, when Hirsch *et al*. developed a Coulter electronic counter, which was used for suspension derived from a known number of cells that were counted manually [9]. Di Girolamo *et al*. developed a technique to determine the number and size of cells in mammals, but it was not a computer method, and it was also done manually by the researchers [10]. With the technological advancements in computer science and embedded systems, some biomedical algorithms and software were developed for biomedical imaging purposes. Chen *et al*. developed computer image analysis for adipose cell size by measuring the cross-sectional area of adipocytes; this method made cell sizing easier for a larger number of cells but was limited to adipocytes only [11]. Björnheden *et al*. developed an automated method in which they took a video of the cell suspension placed between a siliconized glass slide and a cover slip and then acquired images for computer analysis to determine the adipocyte size [12]. Carpenter introduced CellProfiler, which utilized image processing algorithms for automated biological image analysis; the software supported yeast colony counting and classifying, tumour quantification, wound healing assays, and tissue topology evaluation, but the results showed errors when compared to the actual measurements as the number of cells increased [13]. Kamentsky *et al*. released a new version of CellProfiler, CellProfiler 2 aimed at making a pipeline between ImageJ and CellProfiler. To date, it has become a powerful but complex software, which can be difficult to use. To run CellProfiler 2, the researcher needs knowledge of machine learning, a complicated subject for biologists [14]. Osman *et al*. calculated the area of adipose cells by both manual and computerized methods and compared the results. Their computer method, however, was not very accurate as they considered the long and short diameters in cells and considered the cells to be oval; consequently, the determined area of cells was not accurate [15]. Rasband developed the frequently used software, ImageJ, for the National Institutes of Health [16]; this software is used to count and determine the size of adipocyte cells automatically and has additional applications. However, some researchers use ImageJ to perform a manual analysis of the adipocyte size [17]. ImageJ, as one of the most popular software programs in the rapidly growing field of biological image analysis, had a major impact on other software programs [18]. However, ImageJ has some limitations: it is not easy to operate and requires training, especially with more complex problems such as classifying multiple shapes based on their immunostaining.

Several other microscopic image analysers have recently been released, but they generally focus on one aspect of cell analysis and must be used with other software, which increases the time and cost of analysis. Debitage was introduced by Pau to conduct analysis of biological images in cellular phenotypes [19]. This software combines features such as signal processing and machine learning but is limited to a certain type of images and cannot be used widely by researchers without computer education. Also, Sommer *et al*. introduced Ilastik, which is a toolkit for segmentation in analysis of biological high content images; this software can be used only for segmentation purposes [20]. Schindelin *et al*. developed Fiji software for biological image analysis, which is a distribution of ImageJ that has been written in Java language. Fiji is used mostly for cell counting purposes [21], it is not applicable in the colour separation for immuno-stained slides, and the researcher needs to install other software alongside Fiji to operate it. Icy software was developed by Chaumont *et al*. to provide an informatics platform for reproducible biological research [22]; this software provides an excellent combination of a community website and image processing tool, but is mostly intended for researchers who have a significant background in computer programming. Recently, a new version of ImageJ, called ImageJ 2, has been released by Rueden *et al*. [23]; this new version has some major changes such as a new built-in User Interface (UI). As ImageJ is an open source software, it can be easily modified by researchers. However, the improved ImageJ2 still requires training and practice for biomedical researchers, as many lack backgrounds in computer science and programming modification. ImageJ has the features to do cell sizing, however, these features are hidden in menus that take time to learn and use. ImageJ also lacks some other useful and novel analysis features that are provided by our newly developed software, AdipoGauge, as described in this manuscript. Our future goal is to convert AdipoGauge (developed in C++) into an ImageJ Plugin using Java applications.

## 2. Materials, methods, and software devleopment

AdipoGauge (© Moussa lab, 2020) is designed to provide an easy interface for researchers in obesity studies to accurately analyse Haematoxylin & Eosin (H&E) stained images and fluorescently stained images. This software is equipped with different analysing tools to deal with most of the image processing requirements in cell biology. Cell counting and colour separation have been implemented alongside various novel features to provide biology researchers with a powerful, accurate, and user-friendly software, which can be easily applied by investigators with limited computer expertise. The algorithms are described in this section and the results and their accuracy are discussed in the next section, followed by the conclusions and future work to improve the software.

### 2.1. Software development, overview, and images used

**Figure 1.**
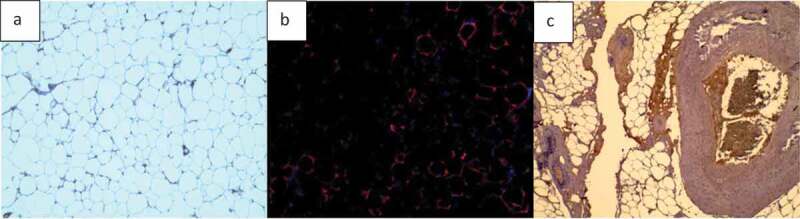
Sample of images used to develop and test AdipoGauge. a) H & E stained mouse adipose tissue image; b) Fluorescently stained macrophages from mouse adipose tissue fixed sections; c) H & E stained human breast tissue section (containing adipose tissue) used for colour separation

Two types of basic microscopic images are analysed in this study: H&E stained images of adipose tissue sections and fluorescently stained images (Figure 1). Adipose tissue histological sections are used to determine the number of cells in the image. Such images do not always have clear borders at the edge of sections, which may appear in different background colours. It is important that in H&E stained adipose tissue images, the clear or unclear borders around the cells be detected accurately, so the number of cells and other required information (i.e. cell size and area histogram) can be acquired with a high level of accuracy.

Fluorescent stained adipose tissue sections are another type of microscopic images that have been considered in this study (Figure 1). The main purpose of using fluorescent stained images is to find the area of interest of each colour in the image by separating the colour channels. Some regions in microscopic images contain valuable information, such as the distribution of adipose tissue or epithelial cell layers of interest. Thus, in fluorescent images, each colour of the image indicates a special part of the cell and knowing the percentage of the marked regions is highly important for bio-researchers. Using simple colour segmentation techniques, the area percentage of a specific organelle can be determined in the image.

### 2.2. Steps for image processing

AdipoGauge uses C++ to enhance microscopic images, perform the required analysis, and generate the desired output for the user who has little or no computer programming skills. Efficient image processing algorithms and a user-friendly graphical user interface (GUI) allow the researcher to analyse different types of images for different objectives with little effort. Generally, microscopic images have RGB colour spaces, and the colour space conversion from RGB channel to other colour spaces is applied when necessary; otherwise, contour detection techniques are implemented to identify the cells. After cell identification, cell counting, cell area evaluation, and histogram generation are performed. Area identification is achieved by filtering out the specified colour channels by the user and comparing the metric distance of objects to determine how similar the objects are. A general overview of the techniques that are used in this framework is provided in subsections: A. Pre-processing, B. Analysing, and C. Post-processing.

It should be noted that one of the most significant improvements to the software is that the user can see changes in the analysis while working with the software; thus, there is no need to repeat the process again and again to see the different results when the image is modified.

#### 2.2.1. Pre-processing

The first type of image introduced in the previous section, the H & E stained adipocyte cell image, reveals significant information, such as the number of the cells and the area of a specific part of the cell organelles. The pre-processing stage is designed to perform a series of image conditioning and calibration steps. The calibration length of the input image is essential in order to generate accurate data; the other important pre-processing step is to identify unwanted objects or information so that it can be excluded from the desired information. Images can also contain noise, such as haze and small black or coloured dots, which should be removed from the image. The pre-processing stage allows the software to enhance results acquired from the laboratory.

##### 2.2.1.1. Calibration

To get the actual quantities such as length and area in micron and micron square, respectively, the image should be calibrated, and a calibration factor utilized. The method used in this software provides the required measurements based on the number of pixels in order to get the most accurate results. For example, cell size can be determined based on the number of pixels the cell contains, and therefore, the method proposed here gives more accurate results compared to other software that does not use pixel counting. The calibration factor shows the length that is assigned to a pixel. Hence, the calibration factor can be evaluated in micron/pixel for the length and micron^2^/pixel for the area measurements.

In AdipoGauge, the calibration can be conducted automatically or manually. Usually, the original output file of the microscope contains the calibration factor as a metadata. The software reads the metadata, if present, in the image file and searches for the micron per pixel value to identify the calibration factor. If the micron per pixel value is not embedded in the image file, the user can enter the value directly into the software. This value can be obtained from the microscope catalogue or the user can determine the pixel length of the calibration line by clicking on the line’s starting and ending points and entering the actual length of the line. The software calculates the calibration factor according to the following formula:

(1) $$\begin{array}{rl} & \;\;\;\;\;\;\;\;\;\;\;\;\;\;\;\;Calibration\;factor\left(MPP\right)\\ & =\frac{length\;of\;the\;calibration\;line\;in\;microns}{length\;of\;the\;calibration\;line\;in\;pixels} \end{array}$$

##### 2.2.1.2. Image enhancement

**Figure 2.**
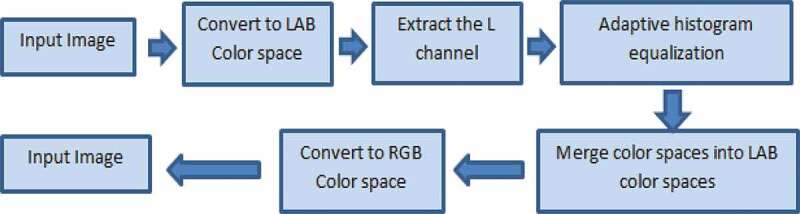
Haze removal process

One of the most important steps in image processing algorithms is image enhancement. As the image is being prepared for photomicrograph, some cells might be damaged; thus, using enhancement methods will lead to more accurate results while analysing the cell images. A similar improvement in results can also be obtained when a specific area of interest is required to be isolated and measured. Moreover, microscopic images might be hazy or noisy due to the preparation process. AdipoGauge is equipped with an algorithm that automatically removes the haze and noise after the user loads the image. The result is a clearer microscopic image, which is more convenient to process. The haze removal process is shown in Figure 2.

##### 2.2.1.3. Loading multiple images

**Figure 3.**
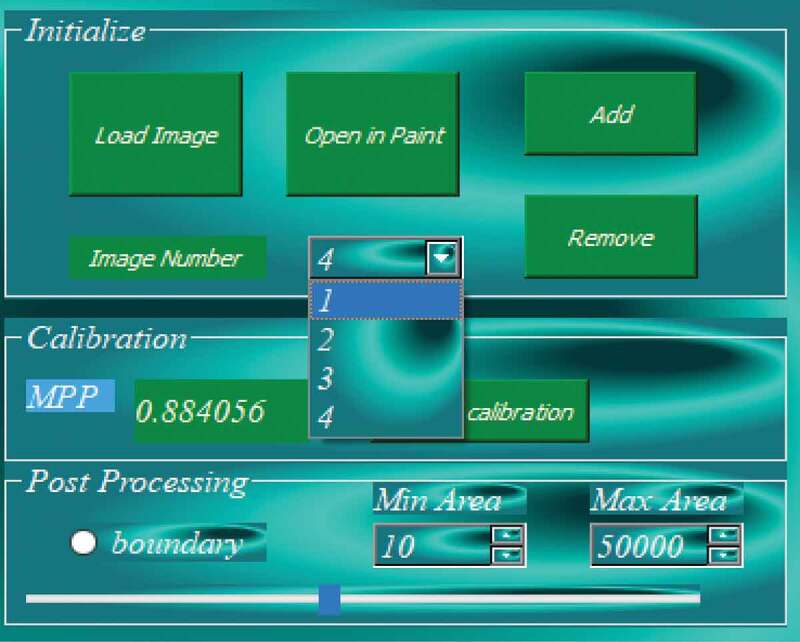
Loading multiple images in software

Another important feature of AdipoGauge is its ability to work with multiple images (Figure 3). This allows the user to have a better control over the image processing and the required results. When a new image is loaded, the program creates a new object for the new image, and as the analysis progresses, every task performed is saved in the object memory, so the user can retrieve the results for multiple images to be used for further comparison.

#### 2.2.2. Analysing

The main objective of AdipoGauge is to detect and analyse the desired features and quantities in microscopic images. After improving their quality, the images are processed in the analysing step, where the user can choose one of the two methods, i.e. cell counting or colour separation.

##### 2.2.2.1. Cell counting

**Figure 4.**
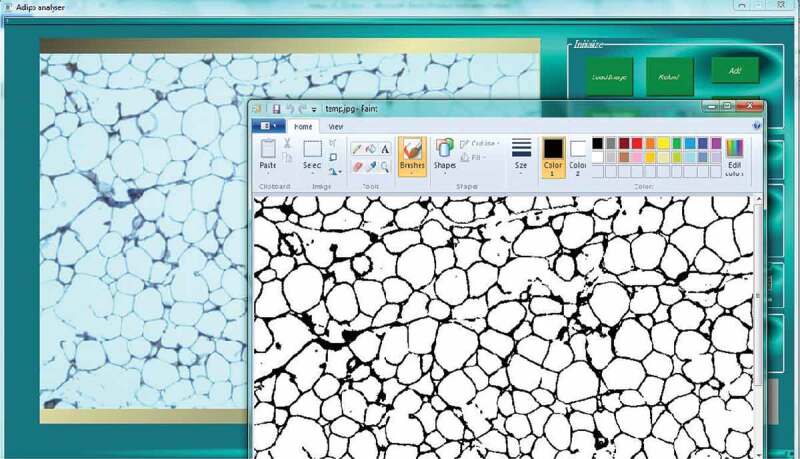
Loading the image in Microsoft Paint to connect the cell border manually

This method is applied to count the number of cells in microscopic images. The H&E stained images are analysed by this algorithm to detect and count the cells. In contrast to previous software that used axes to determine the cells’ areas, AdipoGauge uses contours to identify the required objects (cells). When cell borders are damaged during the photomicrography, the image processing algorithms are often unable to obtain those cells as independent cells. AdipoGauge allows the user to export the images into Microsoft Paint software to perform manual corrections (e.g. connect the cell borders in the images such as light/faint cell membranes) if needed, and then return the images to AdipoGauge (Figure 4). Moreover, the user can see the microscopic image beside the processed image in Microsoft Paint, compare the two images and make the correction accordingly.

In H&E stained images, there are some regions that should be excluded from analysis, such as blood capillaries (depending on the user’s need), or scratches in the slide. AdipoGauge allows the user to exclude objects smaller or larger than a certain area or size by setting the upper and lower limit for the size of cells (i.e. cells smaller than 240 square microns can be ignored). Consequently, any unnecessary objects (small cells, very large cells, skeletal muscle fibres, blood capillaries) can be easily eliminated from analysis and calculations.

**Figure 5.**
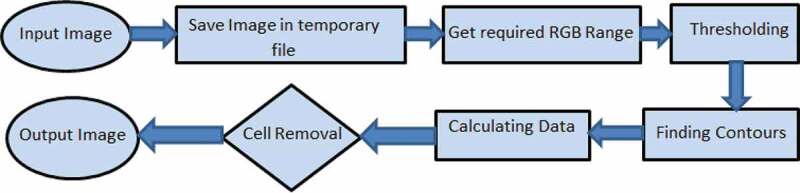
Cell counting flow diagram

Another important feature of AdipoGauge is that it lets the user eliminate from the image any cell or other object and prevent them from being counted by clicking on them; these cells or other objects can be brought back into the calculations by clicking on them again. In medical imaging software, users can specify a range of cell areas to be excluded from analysis. For example, the user can set lower and upper limits for cell counting, and any objects outside this range would be excluded. However, if an unwanted object is of the same size as the cell and cell removal is conducted based on the size range, both the unwanted object and required cells would be excluded from analysis and consequently, the results would be inaccurate. AdipoGauge users can click on an unwanted object and remove it without removing important cells. The counting cell algorithm flow is shown in Figure 5. First, the images are saved in a temporary image location, then the RGB values of the image are determined to do the thresholding, where the threshold is used to find the contours and the cell data. If the user needs to improve results by removing the cells, AdipoGauge will calculate the data for the remaining cells again.

AdipoGauge can exclude incomplete cells (border cells) from being counted or bring them back with one click. This method can be used for H&E stained microscopic images, including images with less clear cell borders, and for unstained cells that have very thin or damaged borders or cells with breaks in their borders. The user can thicken the border lines using a sliding bar. With a greater border thickness, more cells will have continuous borders, and the counting will be more accurate, but it will reduce the calculated cell area. The user can decide on the optimum results and balance between the thickness of the cell borders and the accuracy of the cell number and area with a user-friendly GUI, which shows the effects of the changes at the same time.

##### 2.2.2.2. Colour separation

**Figure 6.**
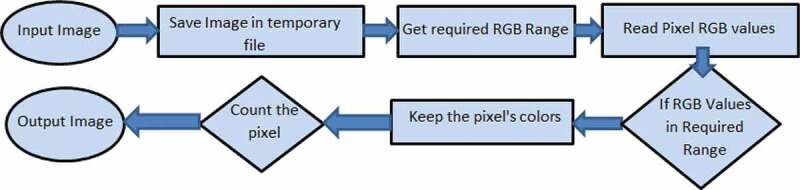
Colour separation flow diagram

Each colour in H&E stained and fluorescent microscopic images represents a different organelle in the cell. The colour separation method provides the user with a wide range of options to separate the desired organelle’s area based on the research needs. In the colour separation algorithm, the user imports an H&E stained image that contains information about cell parts, such as macrophages and nuclei, which is encoded in their colours. With the aid of 3 sliding bars in the GUI of AdipoGauge, users can go through all the colour bandwidths and select the purple, light red, reddish brown or any other colour. After filtering out the desired colours from a specific image, the software calculates the area of interest (areas of the organelles), and the percentage of the separated colours is displayed automatically. The analysed information can be exported into an Excel file. The flow chart for this analysis is shown in Figure 6.

The user can also import a fluorescent image into the software. There are three main colour channels in fluorescent images: red, green, and blue, and these colour channels can be separated by the software. This process is similar to the algorithm for H&E stained images; however, the channels in fluorescent images represent only one colour and the user can see the selected colour on the GUI. This means that if the user chooses the blue channel, only blue would be shown on the GUI and the intensity for all displayed blue will be the same. The user can select each of these three channels or combinations of them and save them to analyse the data. In AdipoGauge, these saved images can be loaded as multiple images, which provides the user with a wider range of analysis tools.

##### 2.2.2.3. Image processing algorithms

The software includes several image processing algorithms to detect the cells and the colour ID of the microscopic images. These algorithms are written in C++ language and involve OpenCV libraries, which have an open source licence. The software can detect the cells by using real-time computer vision.

To find cells in pictures, we considered them as contours. Thus, we use the function called ‘findContours’ in OpenCV. This function, based on the algorithm introduced by Satoshi Suzuki *et al*. in 1985 [24], can retrieve contours from binary images and is a useful tool for shape analysis and object detection. Since we decided to implement this algorithm to count the number of the cells, the images are first converted into binary pictures. Each cell contour is stored as a vector of points. We used ‘CHAIN_APPROX_SIMPLE’ method for contour approximation. We also used ‘RETR_EXTERNAL’ as the mode of contour retrieval. This helps us to get the contours more accurately, as the algorithm does not consider the spaces between cells as contours. The algorithm repeatedly chooses a point inside a white area, which represents a cell, and then finds the neighbouring pixels of the point until it reaches all of the black pixels representing the border of the cell.

The contours are the boundaries of the cells which have the same intensity in binary images; the function ‘findContours’ stores the coordinates of the boundary points of the cells. Thus, for large images, the software requires a large amount of memory, which will slow the computations. To overcome this problem, we have used the ‘CHAIN_APPROX_SIMPLE’ algorithm in OpenCV. In this algorithm, the software stores the coordinates of the beginning and the end point of each line only for each detected contour. Hence, the amount of required memory decreases significantly for the software. The algorithm used in the software is remarkably fast for large images and can be run on computers with less processing power and memory. As a result, the researcher can reduce time and expense to analyse a microscopic image.

For the colour separation, we used the C++ language and OpenCV libraries to analyse the images. We saved the images in Mat format in C++ and then the intensity of each colour channel (blue, green, and red) was determined and stored in a vector. This vector was used to separate the colours of each image.

#### 2.2.3. Post-processing

After analysing the images, the acquired data can be displayed in the interface or stored, based on the needs of the user. The post processes include generating area size histograms, black and white cell maps, separated colour maps, area of interest maps, desired area size and its percentage, removing or counting the bordering cells of the image, and removing the unwanted objects by clicking on them and exporting the data into an Excel file.

## 3. Results

### 3.1. Calibration and object identification

#### 3.1.1. Automatic calibration

**Figure 7.**
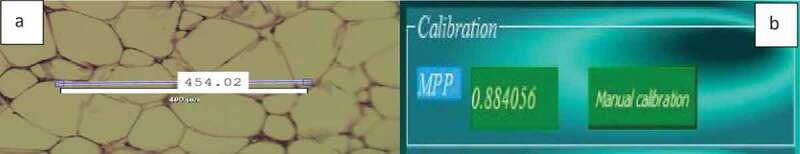
Adipose tissue histology section for image calibration. a) Manual calibration b) Automatic calibration

The developed software enables automatic as well as manual calibration. After importing the image in Figure 1(a) into the software, the calibration factor was automatically detected as 0.8840 microns per pixel. The researcher can also use the scale bar to calculate the calibration factor manually; this can be done by sketching the sliding bar over the calibration guide on the microscopic image and then inserting the actual length on the image. The calibration factor yields (400/454.02) = 0.8810 MPP, which approximately equals the value assigned to the image.The manual and automatic calibrations are shown in Figure 7(a,b) respectively.

#### 3.1.2. Object identification

**Figure 8.**
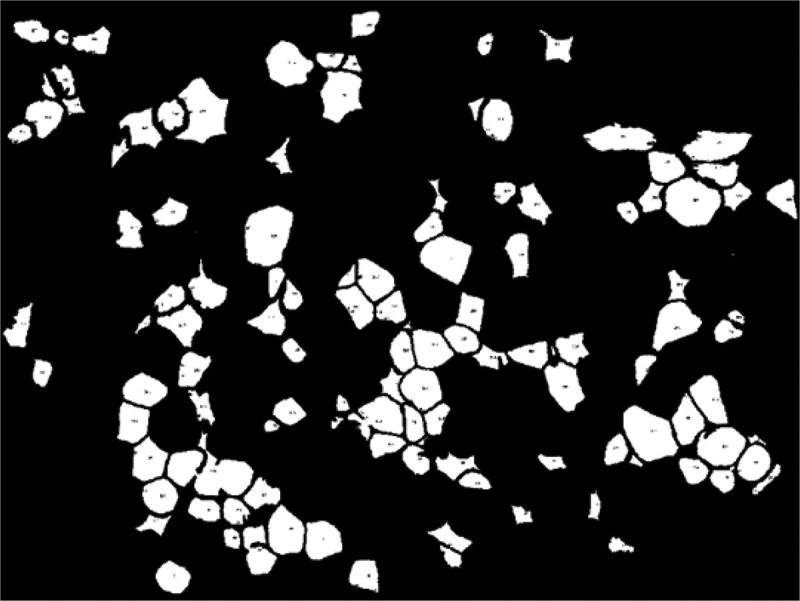
Adipose tissue histology section

AdipoGauge is equipped with the tools to identify the objects with different criteria. Figure 8 shows different objects that have been selected from the image in Figure 1(a). The criterion for these objects is the size of the cells. In this image, the cells with a size between 250 and 2000 square microns have been displayed.

Objects with area between 250 and 2000 square microns.

**Figure 9.**
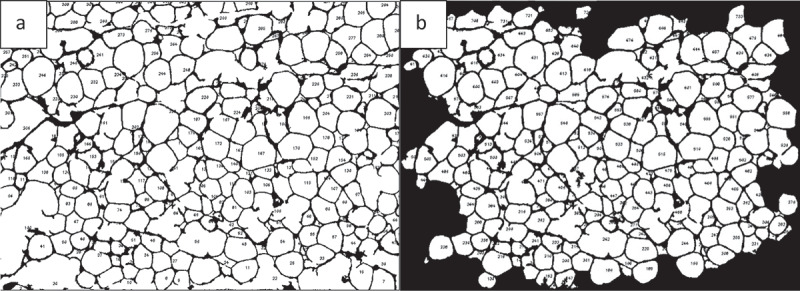
Adipose tissue histology sections for cell measurements. a) Image includes border cells b) Image without border cells

Another criterion that can be specified in AdipoGauge is removing the bordering or incomplete cells. Figure 9(a,b) show the images with and without border cells; the user can define the objects required for the research purposes and assign a number to each cell.

**Figure 10.**
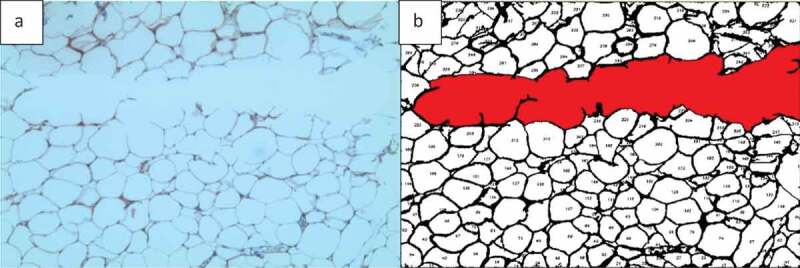
Adipose tissue H&E stained section. a) Image with a scratch b) Segmented cells image in which the scratch is excluded from the analysis

AdipoGauge can remove the unwanted objects, such as defects, background, scratches in specific areas of the slide, or blood vessel sections. It gives the researcher the freedom to eliminate any unwanted object without the need for specific criteria. For example, if the researcher wants to remove an object but does not want to impose a limit on its size or does not want to delete other objects similar to the unwanted object, the user can simply click on it to exclude it from the calculations and bring it back by clicking on it again. Figure 10(a,b) show this feature.

This is a very important feature of AdipoGauge. In other software packages, including ImageJ, if the user removes an object and they want to bring it back, they have to close the program, reload the image, and start all over again; thus, it is very time consuming. However, in AdipoGauge one can eliminate or bring back objects in real time without closing the program, as many times as needed. Moreover, in ImageJ, the user can save each step, but if they need to proceed differently, they must redo the whole process. This problem is solved in AdipoGauge as the user can go back through the steps without closing the program and restarting it again.

### 3.2. Applications: cell counting/sizing and colour separation

#### 3.2.1. Cell counting/sizing

**Figure 11.**
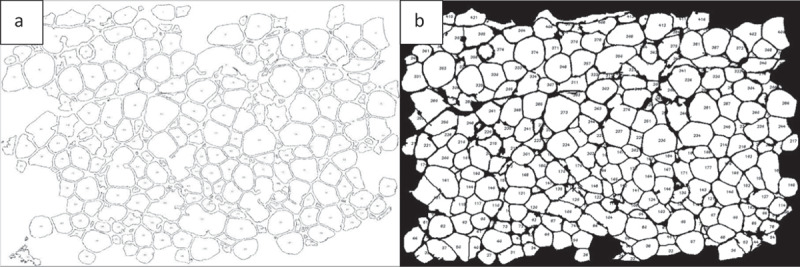
Excluding border cells and output of Image 1A analysis. a) Cells counted by ImageJ. b) Cells counted by AdipoGauge after manual processing of the image using Microsoft Paint

AdipoGauge uses algorithms to analyse the number of cells and their areas. The number assigned to each cell is represented on the cell image in the GUI, as well as in the Excel file that includes the area of each cell. The cell counting analysis shows that the image in Figure 1(a) has 259 cells and their area is 368,325.0 square microns without the border cells. To compare the results, the same image was processed in ImageJ which shows that there are 266 cells and the area is 368,643.8 without border cells. In this analysis the difference in the number of cells is only 7 cells, 2.6%, and the difference in the area is 318 square microns or 0.09%, which demonstrates that AdipoGauge is as accurate as ImageJ, without correcting the image with Microsoft Paint. When the image is further processed in AdipoGauge using Microsoft Paint (integrated into the program), the results become more accurate as the user can identify boarders better and delete unwanted objects. In this case, the software counted 294 cells and the area is 407,925.3 square microns (Figure 11(a,b)). Thus, the segmentation and cell counting are significantly improved, and the cells are well defined by AdipoGauge. Data analysis for ImageJ and AdipoGauge before and after manual corrections using Microsoft Paint is given in Table 1. Statistical correlations between the two software packages are provided in the section on the statistical analysis.

**Table 1.** Data analysis (cell number and size) for adipocytes in Figure 11 by ImageJ and AdipoGauge

	ImageJ Analysis		AdipoGauge Analysis Before correcting by Paint		AdipoGauge Analysis After correcting by Paint	
Area Bins (Sq. Micron)	Number of Cells	Area (Sq. Micron)	Number of Cells	Area (Sq. Micron)	Number of Cells	Area (Sq. Micron)
10–250	70	6268.5	68	6151.6	59	6227.6
251–500	24	9368.8	24	9321.8	29	11,211.2
501–1000	45	34,186.9	43	33,453.0	52	40,512.5
1001–2000	61	92,375.8	57	87,010.5	70	103,242.5
2001–3000	33	80,366.1	34	83,795.1	54	128,670.5
3001–4000	18	61,043.3	18	61,054.1	21	72,685.9
4001–6000	10	47,027.7	10	48,241.9	10	45,375.1
6001-	5	38,006.5	5	39,297.0	0	0
**Total**	266	368,643.8	259	368,325.0	294	407,925.3

##### 3.2.1.1. Histogram of results

**Figure 12.**
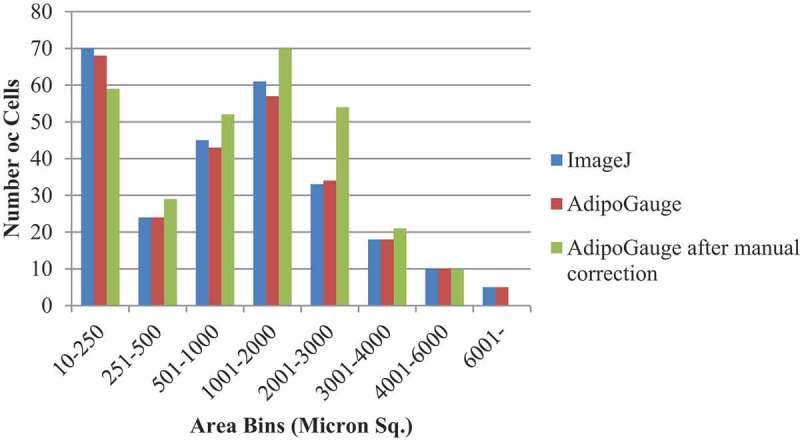
Histogram of area of adipocytes from. Figure 1(a)

AdipoGauge can be used for further analysis of cell images. The data stored in an Excel file that contains the information about cells and their areas can be used to draw the histogram of cells based on their size or other features. Figure 12 shows the number of cells for Figure 1(a) sorted in 8 bins. It can be inferred from the histogram that ImageJ and AdipoGauge can detect the cells very well. However, the results become more accurate after correcting the cell borders (not very clear in the original image) in AdipoGauge using Microsoft Paint; the number of small and big cells is reduced and the number of medium-sized cells is increased. The reason for this is that in automatic detection by ImageJ and AdipoGauge cells that are not well defined are considered to be one cell; when the image is processed by Microsoft Paint, the borders become clear and AdipoGauge can detect separate cells.

#### 3.2.2. Colour Separation

**Figure 13.**
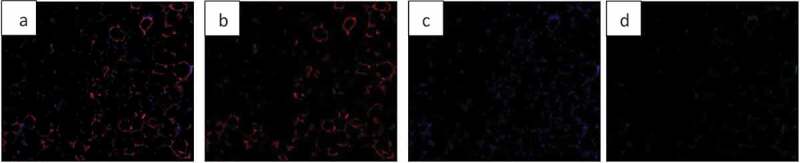
Adipose tissue histology sections a) Fluorescently stained image b) Red channel c) Blue channel d) Green channel

**Figure 14.**
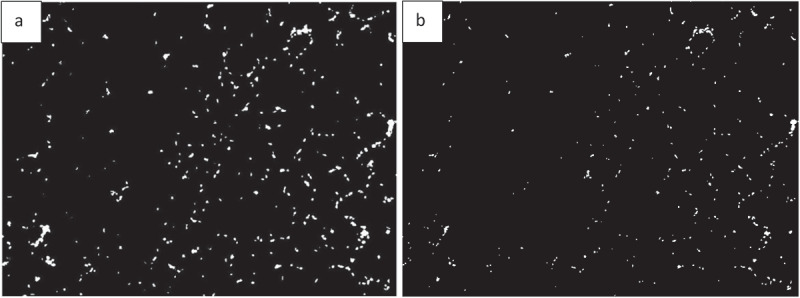
Adipose tissue sections: Image conversion into black & white to enhance visibility. a) Blue area of Figure 13(c) (nucleus). b) Green area of Figure 13(d)

Finding the area occupied by different colours in an image is possible with AdipoGauge. It is utilized for two types of images; H&E stained images and fluorescent images. The researcher can split the three main colour channels (red, green, and blue) of a fluorescently stained image (Figure 13(a)) to display them separately, as shown in Figure 13(b-d). The images for blue and green channels in Figure 13(c,d) are converted into a black and white image (Figure 14(a,b) for more visibility.

Researchers can combine two channels as well. Thus, we have a total of 6 colour channels including red, blue, green, red and blue, red and green, and blue and green.

**Figure 15.**
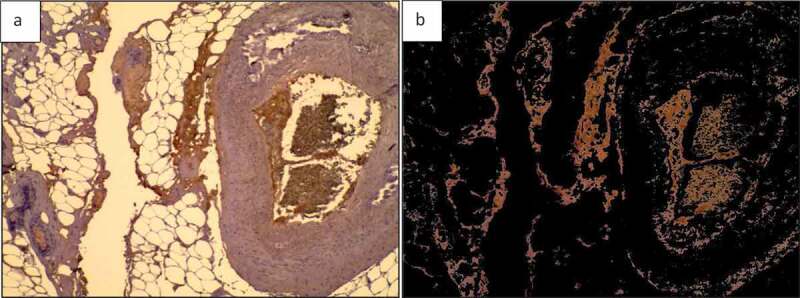
Breast tissue H&E stained section. a) H & E stained image; b) Specified colour range ‘reddish brown’ is separated out

A colour separation algorithm is used to determine the reddish-brown area of Figure 1(c) and the result is displayed in Figure 15. In Figure 15(b) the area of reddish-brown colour is calculated as 10.21% of the whole image in Figure 15(a).

### 3.3. Statistical analysis of output data from AdipoGauge and ImageJ

We used linear regression to determine correlation coefficients and their p values for cell size and cell number measurements, conducted using AdipoGauge and ImageJ.

**Figure 16.**
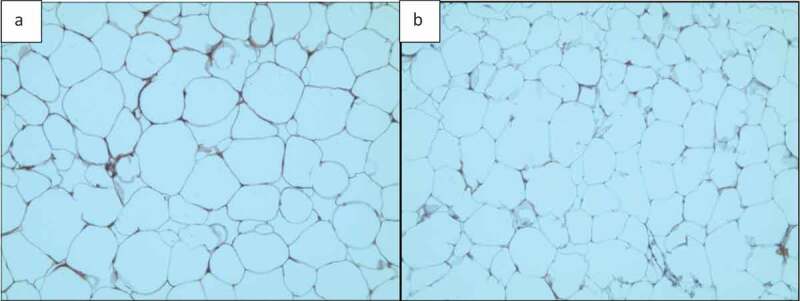
Adipose tissue H&E stained sections. a) Section with clear cell membranes/borders b) Section with low quality cell membranes/borders

Two adipose tissue histology images, shown in Figure 16(a,b), were selected for this purpose. Both images were analysed by ImageJ and AdipoGauge, and the border cells were counted as well. Since the cells in Figure 16(a) are relatively large with well-defined borders while cell borders in Figure 16(b) are not very clear, the images were specifically chosen to compare the two software packages.

#### 3.3.1. *Analysis of Adipose Tissue Sections with Clear Cell Borders (**Figure 16(a*, *Figure 17*, *and* *Table 2*)

**Figure 17.**
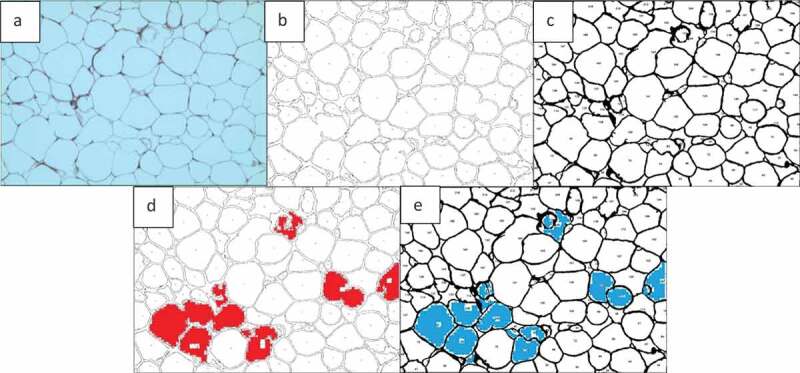
Output of cell counting results for. Figure 16(a). a) Original Image of adipose tissue histology. b) ImageJ image. c) AdipoGauge image. d) Cells that were counted differently in ImageJ in red. e) Cells that were counted differently in AdipoGauge in blue

In Figure 16(a) the total number of cells was counted as 135 using ImageJ and 144 using AdipoGauge after correcting the cell borders using Microsoft Paint (to mark light cell membranes/borders). Due to corrections AdipoGauge detected poorly defined cells. Consequently, the number of medium-sized cells increased while the number of big cells decreased in AdipoGauge analysis vs. ImageJ analysis. The output cell counting results are displayed in Figure 17(b,c) for ImageJ and AdipoGauge, respectively. We highlighted in red and blue the cells that were counted differently by the two software packages (Figure 17(d,e)).

**Table 2.** Data analysis for Figure 17(a) by ImageJ and AdipoGauge

	ImageJ Analysis		AdipoGauge Analysis After correcting manually		Difference in Area (%)
Area Bin (Sq. Micron)	Number of Cells	Area (Sq. Micron)	Number of Cells	Area (Sq. Micron)	
10–500	34	5897.6	36	7577.2	28.4
501–1000	12	7938.6	14	9496.3	19.6
1001–2000	14	20,291.5	16	22,828.2	12.5
2001–4000	22	64,989	24	70,088.1	7.8
4001–6000	18	84,622.5	20	96,894.5	14.5
6001–8000	15	104,020.6	16	110,982.7	7.0
8001–10,000	7	59,847.7	8	69,011.4	15.3
10,001-	13	182,616.7	10	131,721.0	27.9
**Total**	135	530,224.3	144	518,599.5	2.1

We also counted the total area of cells by ImageJ as 530,224.3 square microns and by AdipoGauge as 518,599.5 square microns. The difference is ~2.1% (Table 2).

Statistical analysis was used to verify the accuracy and correlation of results from ImageJ and AdipoGauge data. Linear regression in Excel was used to determine the correlation coefficient (R) for both cell size and number, based on data in Table 2. P values indicate strength and statistical significance of this correlation.

For the cell number, the correlation coefficient between the two software packages is R = 0.982815341 (p = 0.0000125), indicating that the results for both software packages are very similar with a strong correlation. For the cell area, R = 0.950701594 (p = 0.000288563) indicates 95% similarity between the data obtained with the two software packages.

#### 3.3.2. *Analysis of adipose tissue sections with unclear cell borders (**Figure 16(b*, *Figure 18*, *and* *Table 3*)

**Figure 18.**
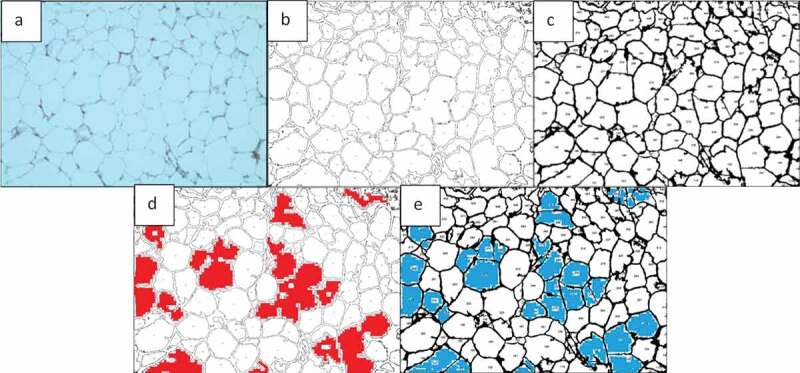
Comparison of output results for image in. Figure 16(b). a) Original Image of adipose tissue histology. b) Image analysed by ImageJ. c) Image analysed by AdipoGauge. d) Cells counted differently in ImageJ. e) Cells counted differently in AdipoGauge

Tissue section image (Figure 18(a)) was analysed by both software packages. In this image, the borders are not very clear and there are very thin cell membranes. Using ImageJ, the total number of cells was counted as 133 cells (Figure 18(b)). Some of the cell borders were not detected correctly, and consequently the objects were counted as one merged cell (Figure 18(d)). Table 3 shows that the number of medium-sized cells is lower than the actual value while there is an increase in the number of large cells that consist of more than one cell. This problem was solved in AdipoGauge as we corrected the cell borders manually using Microsoft Paint and then we counted the cells with AdipoGauge (Figure 18(c,e)). The number of cells increased to 179, and the difference is about 35%. The total area of cells was calculated as 475,217.7 vs. 490,921.3 square microns by ImageJ and AdipoGauge, respectively; the difference is ~3.3%. Cells that were counted differently are indicated in red and blue for ImageJ and AdipoGauge, respectively (Figure 18(d,e)).

**Table 3.** Data analysis for Figure 18(a) by ImageJ and AdipoGauge

	ImageJ Analysis		AdipoGauge Analysis After correcting manually		Difference in Area (%)
Area Bin (Sq. Micron)	Number of Cells	Area (Sq. Micron)	Number of Cells	Area (Sq. Micron)	
50–500	34	7341.6	45	8766.6	19.4
501–1000	13	9349.9	19	13,715.2	46.7
1001–2000	20	29,427.6	31	45,496.1	54.6
2001–4000	27	76,285.2	36	103,826.7	36.1
4001–6000	16	87,302.8	24	121,763.6	39.5
6001–8000	10	67,072.4	15	105,405.8	57.2
8001–10,000	3	26,700.0	4	33,830.9	26.7
10,001-	9	171,738.2	5	58,116.4	66.1
**Total**	133	475,217.7	179	490,921.3	3.3

The first row of Table 3 shows that ImageJ detected 34 cells and AdipoGauge detected 45 cells. This indicates that some cells with broken shared borders/membranes were considered as one cell in ImageJ, which accounts for a lower cell number and bigger cell size. By using AdipoGauge and opening the image in Microsoft Paint, those broken or unclear/thin cell border lines are corrected manually, and as a result, the number of cells is counted correctly. Therefore, fewer small cells were placed in the bin with cell areas between 50 and 500 square microns for ImageJ. Overall, the calculated area for this bin by ImageJ is 7341.6 square microns and by AdipoGauge is 8766.6, which was expected due to a greater number of cells detected by AdipoGauge. Similar comparisons can be made for other bins with different cell sizes. Overall, AdipoGauge detects more cells, which can be verified by visual observation and hand counting of cells in the original H&E image.

The last row in Table 3, showing the number of cells bigger than 10,000 square microns, confirms the above statements. Since ImageJ is unable to automatically correct unclear cell borders between cells, it detected 9 big cells, while this number is only 5 for AdipoGauge. Thus, there is a bigger difference between the two software packages (66%) for very large cells, compared to 19% for the smallest cells.

Statistical analysis was used to verify the accuracy and correlation of results from ImageJ and AdipoGauge data. Linear regression in Excel was used to determine the correlation coefficient (R) for both cell size and number, based on data in Table 3. P values indicate strength and statistical significance of this correlation.

For the cell number, the correlation coefficient between the two software packages is R = 0.97372 (p = 0.00004450), while for the cell area, R = 0.52901 (p = 0.17762). Thus, cell numbers evaluated using both software packages are very similar (~97.4%), with a strong correlation. However, for the area of the cells, the similarity between the data obtained with the two software packages is only ~53%. This indicates that when cell borders are not clear in the image, using AdipoGauge integrated with Microsoft Paint can significantly improve the accuracy of the results.

#### 3.3.3. Analysis of immunofluorescent images of macrophage staining in adipose tissue

**Figure 19.**
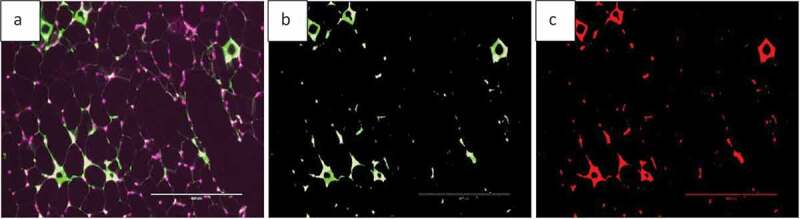
Fluorescent image of macrophage staining in adipose tissue of obese mice fed a high-fat diet. (a). Original Image (b). Image analysed for macrophage detection from AdipoGauge. (c). Analysis result from ImageJ

Fluorescent images can be analysed by AdipoGauge and different parts of the image can be separated based on their colour to demonstrate different cell organelles. To illustrate this application, we used images of adipose tissue of obese mice fed a high-fat diet. The mice had an increased level of macrophages in adipose tissue (Figure 19(a)); the area of macrophages is shown in green. The image was analysed by AdipoGauge and ImageJ, and the results are shown in Figure 19(b,c), respectively.

**Figure 20.**
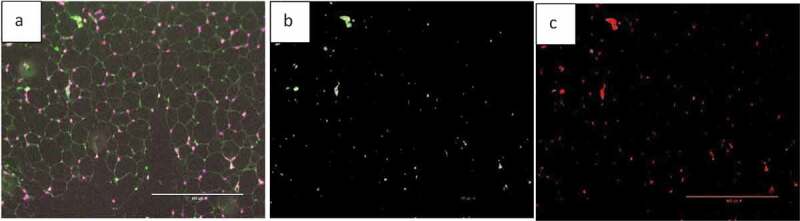
Fluorescent image of macrophage staining in adipose tissue of obese mice fed a high-fat diet supplemented with fish oil (using galectin). (a). Original Image (b). Image analysed using AdipoGauge. (c). Analysis using ImageJ

In addition, we also used images of adipose tissue of mice fed a high-fat diet supplemented with fish oil, which is an anti-inflammatory food component known to reduce macrophage content in adipose tissue [17]. Figure 20(a) shows an EPA fluorescent slide and the area of the macrophage (green colour). The image was analysed by AdipoGauge and ImageJ, and the results are shown in Figure 20(b,c), respectively.

We compared the area of macrophages in adipose tissue for Figures 19a and 20(a), calculated by ImageJ and AdipoGauge, and the data are shown in Table 4. The images from obese mice fed a high-fat diet have a high level of macrophage staining, and the estimated area is comparable between ImageJ and AdipoGauge (0.36% difference). However, for the fish oil supplemented group, with very few macrophages detected, the difference between the two software packages is ~24%; and this is likely because even though the difference in percentage is relatively high, but the calculated area by both software are very small and close to each other.

**Table 4.** Data analysis for area of interest (Macrophage) by ImageJ and AdipoGauge

Image Name	Macrophage area ImageJ (%)	Macrophage area AdipoGauge (%)	Difference in Area (%)
High Fat (19A)	2.81	2.82	0.36
High Fat supplemented with fish oil (20A)	0.80	0.61	23.75

## 4. Discussion

Biomedical researchers are increasingly in need of improved tools to evaluate their experimental data, and especially images such as those generated from histology or immunostainings. In obesity research, there is a critical need for automated tools to assess changes in the cell size, number and/or to quantify other cell changes.

Our primary goal was to develop software to specifically help obesity researchers to accurately and easily analyse adipose tissue images, for the purpose of counting fat cells and determining their size and quantifying stained cells or proteins in tissue sections.

The accuracy of results was verified by statistical analysis. Several images were analysed, and the number of their cells was counted by AdipoGauge and ImageJ. As shown above, AdipoGauge produced better results than ImageJ.

While others have reported various means to assess cell sizing, very few have developed automated means to analyse immunofluorescent-labelled sections of adipose tissue. Our software addresses many drawbacks of the currently used tools for cellularity measurements. Most existing methods are unable to process unstained cells, whereas AdipoGauge can process unstained as well as stained slides (H & E, fluorescence staining or chromogenic staining). Most available tools are mainly used to count the number of cells, and do not offer the option of analysing areas of interest such as areas in H&E or other stained slides that the user can separate by colours. Moreover, these tools fail to identify cell boundaries when they are not well defined in the histology section or when sections are of poor quality. The exclusion of damaged cells and border cells is among the most important functions provided by AdipoGauge. Equally important is the user-friendly and well-developed interface that gives the researcher the option of choosing which cells to include or not in the overall analysis. Another innovative feature of this novel tool is that it enables the study of specific areas of interest, such as cell nuclei or macrophage quantification. In addition, the software merges applications for obesity researchers into one package, which can be further customized as needed.

Future work will include implementing an algorithm to read a video as input. This can be done by extracting images from the video, analysing them, and then combining them as a video file. We would also like to develop a tool to allow this software to interact with cellphone operating systems and use the cellphone’s camera/video to take a picture, insert it into the program, and analyse it automatically.

## 5. Conclusions

AdipoGauge is intended to be widely used in laboratories focused on obesity research, but can also be expanded to other areas, as any cell or tissue section can be analysed by this software. AdipoGauge is an easy-to-use package that provides a wide range of analytical tools specifically designed to study obesity and adipose tissue and cells. The software is time efficient and accurate. Since analysing the data with AdipoGauge can be easily and quickly done in any laboratory, it will be beneficial for practitioners as well as other users in medical institutions where rapid patient image analyses are needed. Overall, AdipoGauge is an excellent tool for bio/medical researchers to help improve the outcomes and efficiency of their practice or research.

## Note

The software AdipoGauge (© Moussa lab, 2020) can be provided to academic researchers by contacting the corresponding author, Dr. Hanna Moussa (hanna.moussa@ttu.edu), and request the downloadable link of the software with the following expectations: 1. The person who wishes to use the software for their research work, must cite this original publication of the software.

2. The software is provided “as is”, without warranty of any kind, expressed or implied, including but not limited to the warranties of merchantability, fitness for a particular purpose and noninfringement.
